# Risk of Bias in Iranian Randomized Trials Included in Cochrane Reviews

**DOI:** 10.34172/aim.2022.61

**Published:** 2022-06-01

**Authors:** Ali Kabir, Ahmad Sofi-Mahmudi, Arman Karimi Behnagh, Vahid Eidkhani, Hamid Reza Baradaran, Payam Kabiri, AliAkbar Haghdoost, Bita Mesgarpour

**Affiliations:** ^1^Minimally Invasive Surgery Research Center, Iran University of Medical Sciences, Tehran, Iran; ^2^Cochrane Iran Associate Centre, National Institute for Medical Research Development (NIMAD), Tehran, Iran; ^3^Endocrine Research Center, Institute of Endocrinology and Metabolism, Iran University of Medical Sciences, Tehran, Iran; ^4^Department of Epidemiology and Biostatistics, School of Public Health, Tehran University of Medical Sciences, Tehran, Iran; ^5^Social Determinants of Health Research Centre, Institute for Futures Studies in Health, Kerman University of Medical Sciences, Kerman, Iran

**Keywords:** Cochrane, Evidence-based medicine, Iran, Randomized controlled trial, Systematic review

## Abstract

**Background:**

Among interventional studies, randomized controlled trials (RCTs) provide the highest level of evidence. However, RCTs can be susceptible to the risk of bias (RoB). Systematic reviews can be performed to appraise RoB in the included articles using evaluative tools. This study aimed to describe the main characteristics and focus on the RoB of RCTs conducted in Iran and included in Cochrane Reviews (CRs).

**Methods:**

We searched "Iran" by selecting the "Search All Text" and "Review" fields in the *Cochrane Database of Systematic Reviews* within Ovid. CRs that included the RCTs conducted in Iran were retrieved. A trial was selected only if it was included in CRs, described as a controlled clinical trial, involved human subjects and CR authors assessed its RoB. The trials were characterized by investigating the relevant articles and the table "Characteristics of included studies" in each CR. The RoB was investigated by collecting the review authors’ judgments based on RoB assessment tables in the CRs.

**Results:**

Out of 1166 Iranian RCTs included by 571 CRs, low RoB was found in 44.9% for random sequence generation, 20.8% for allocation concealment, 32.3% for blinding of participants/personnel, 36.5% for blinding of outcome assessors, 56.3% for incomplete outcome data, 41.3% for selective outcome reporting and 53.8% for other sources of bias.

**Conclusion:**

The RoB in Iranian RCTs was found to be mostly high or unclear. While this is similar to the global situation, it is recommended that the methodological quality of conducting and reporting RCTs be addressed in Iran.

## Introduction

 Bias or systematic error can lead to over-reporting or under-reporting the treatment effects. Although randomized controlled trials (RCTs) are considered the gold standard for designing clinical research, their design, conduct, analysis and reporting are frequently at risk of flaws.^1^ Moreover, results of systematic reviews, especially those focusing on interventional studies and including RCTs, mainly depend on the quality of RCTs.^2^ Including low-quality RCTs in a systematic review can result in unreliable estimates of the effects.^3^ In the past decades, efforts made to improve the quality of RCTs include developing the Consolidated Standards of Reporting Trials (CONSORT).^4^ Failing to observe these standards by journals or incomplete adherence to the guidelines after their adoption, providing inadequate training for researchers and poor practices and processes of research governance in place can influence the quality of conducting and reporting in trials.^5-8^

 The quality of RCTs has been frequently investigated in terms of the journal,^9,10^ the subject,^11,12^ the publication year ^13^ and rarely by the country.^14,15^ These studies have assessed the quality of reporting in trials mainly using the CONSORT statement and therefore relied on the reports’ information.

 Cochrane reviews (CRs) are considered the gold standard for systematic reviews owing to their implementation of the most stringent standards for quality assessment in terms of conducting and reporting.^16^ CRs are published in the CDSR as the most distinguished journal and database of systematic reviews and meta-analyses in healthcare. As part of the Cochrane Library, the CDSR includes all CRs and protocols prepared by authors who register titles with a CR group.^17^ Each CR group supports CR authors in methodological and editorial issues by focusing on a specific topic. The original Cochrane risk of bias (RoB) tool recommended for the included RCTs in 2008 was updated in 2011 and revised in 2019.^2,18^

 The number of RCTs registered in the Iranian Registry of Clinical Trials (IRCT), established as a WHO primary registry at the end of 2008,^19^ substantially rose to more than 25 400 in July 2020. The quality of reporting in Iranian RCTs has been investigated previously^14,20-23^ and it is shown that the quality of RCTs conducted in Iran might be suboptimal.

 To the best of the authors’ knowledge, this quality has not been evaluated yet in terms of RoB. This evaluation can also help rank countries in terms of the quality of their RCTs. This study was conducted to provide an overview of the characteristics and RoB in the RCTs conducted in Iran and included in CRs and to identify the areas requiring improvements the most.

## Materials and Methods

 We searched “Iran” by selecting the “Search All Text” and “Review” fields in the CDSR within Ovid on September 30, 2019. CRs were screened to identify those that included Iranian RCTs. The main characteristics extracted of the eligible CRs included DOI, publication year, CR Group, numbers of the included trials and Iranian trials and the total population of the included trials. A trial was selected only if it was included in CRs, described as a controlled clinical trial, conducted in Iran, involved human subjects and CR authors assessed its RoB.

 Four independent authors, namely AK, AKB, VE and AS, extracted each trial’s characteristics by investigating the table “Characteristics of included studies” in each CR and the relevant published article in case the provided data were insufficient. The collected data associated with the features of the trials included the first author’s name, the article’s title, language and publication year, the name of the journal, the type and beginning and end dates, city and province of the intervention, the number of centers involved, the sample size, the allocation type and RoB assessment results. RCTs, quasi-experimental studies, crossover RCTs, cluster RCTs, non-randomized and others were considered RCT assignments. The allocation type was classified as treatment, supportive care, prevention, diagnostic, and patient education following reviewing 10% of each CR group’s records. Treatment was defined as any pharmaceutical intervention, and supportive care was considered any other healthcare interventions from psychologists, rehabilitation, physiotherapy, occupational therapy, dietetics, complementary therapies as well as pain specialists and social workers.^24^

 CRs appraise RoB in the included RCTs using a specific domain-based evaluative tool. RoB was judged in each criterion as ‘Low risk’, ‘High risk’, or ‘Unclear risk’ when there was lack of information or uncertainty over the potential for bias. To describe RoB assessments, the review authors’ judgments were extracted for each criterion as an RoB assessment table in the CRs. The standard format of Cochrane RoB assessment comprises random sequence generation, allocation concealment, blinding of participants and personnel (performance bias), blinding of outcome assessors (detection bias), incomplete outcome data (attrition bias), selective outcome reporting and other sources of bias. An aggregate assessment was performed for the blinding reported in other formats such as subjective and objective outcomes or assessor, analyst, participants and/or caregivers. The blinding category was considered low ROB if all subcategories of blinding items were reported as low ROB and it was considered as high ROB if one or more subcategories blinding items were reported as high ROB. Otherwise, the blinding bias was reported as unclear. The diverse types of bias in each CR reported as funding bias, intention-to-treat bias, sample-size bias, for-profit bias and power calculation bias were aggregated as other sources of bias where appropriate. The RoB trend in Iranian RCTs was examined to explore the role of time in improving methodology and reporting. The present study did not assess the overall RoB, given that this tool does not recommend evaluating this risk.

###  Statistical Analysis

 The extracted data were analyzed in R version 3.6.0 (2019-04-26) (R Foundation for Statistical Computing, Vienna, Austria; http://www.R-project.org). The RCTs were characterized using frequency and relative frequency.

## Results

**Figure 1:**
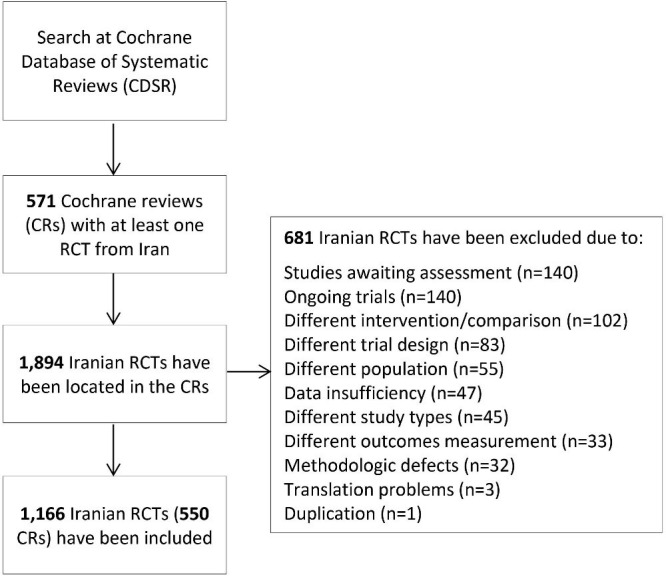
Flow Diagram of Search for Iranian RCTs in Cochrane Reviews.

 The RoB had been assessed for 1166 out of 1894 Iranian RCTs included in 571 retrieved CRs. The RCTs in the retrieved CRs were excluded mainly due to being an ongoing type of trial (20.6%) or their assessment being pending (20.6%) (Figure 1).

**Figure 2:**
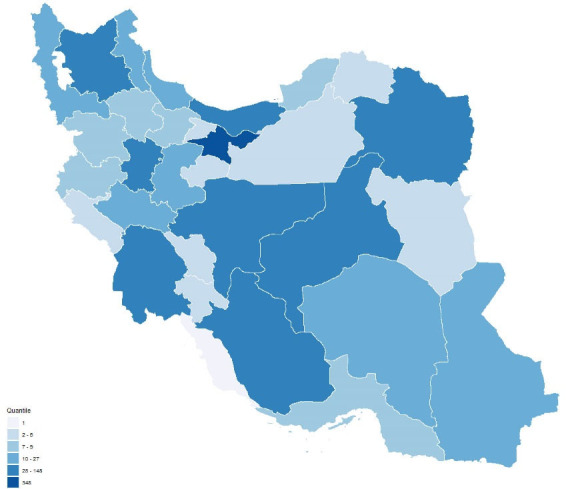
Distribution of conducting sites of Iranian RCTs. Plot created using R package version 3.6.0 (2019-04-26) (R Foundation for Statistical Computing, Vienna, Austria; http://www.R-project.org).

 A total of 7.3% (2.7% of the population) of 15 894 RCTs recruiting an entire population of 5 461 452 and included in the CRs were conducted in Iran and their RoB had been assessed. Five CRs with an unknown population were, however, excluded from the analysis. Fifty CR groups produced 63.7% of the CRs from 1970 to 2018 after 2013 (Figure 2). The majority of Iranian RCTs addressed pregnancy and childbirth (n = 250, 21.4%), gynecology and fertility (n = 158, 13.5%) and skin and oral health (n = 70, 6.0%). A CR entitled “Interventions for old world cutaneous leishmaniosis” by members of the Cochrane Skin Group in Madrid published in 2017 included the highest number of Iranian RCTs, i.e. 27 out of a total of 49.^25^ No Iranian RCTs were reviewed by Cochrane HIV/AIDS, Lung Cancer, Methodology, STI and Urology groups. Two Iranian RCTs were included in two CRs and the RoB assessment performed in one of them was inconsistent.

**Figure 3:**
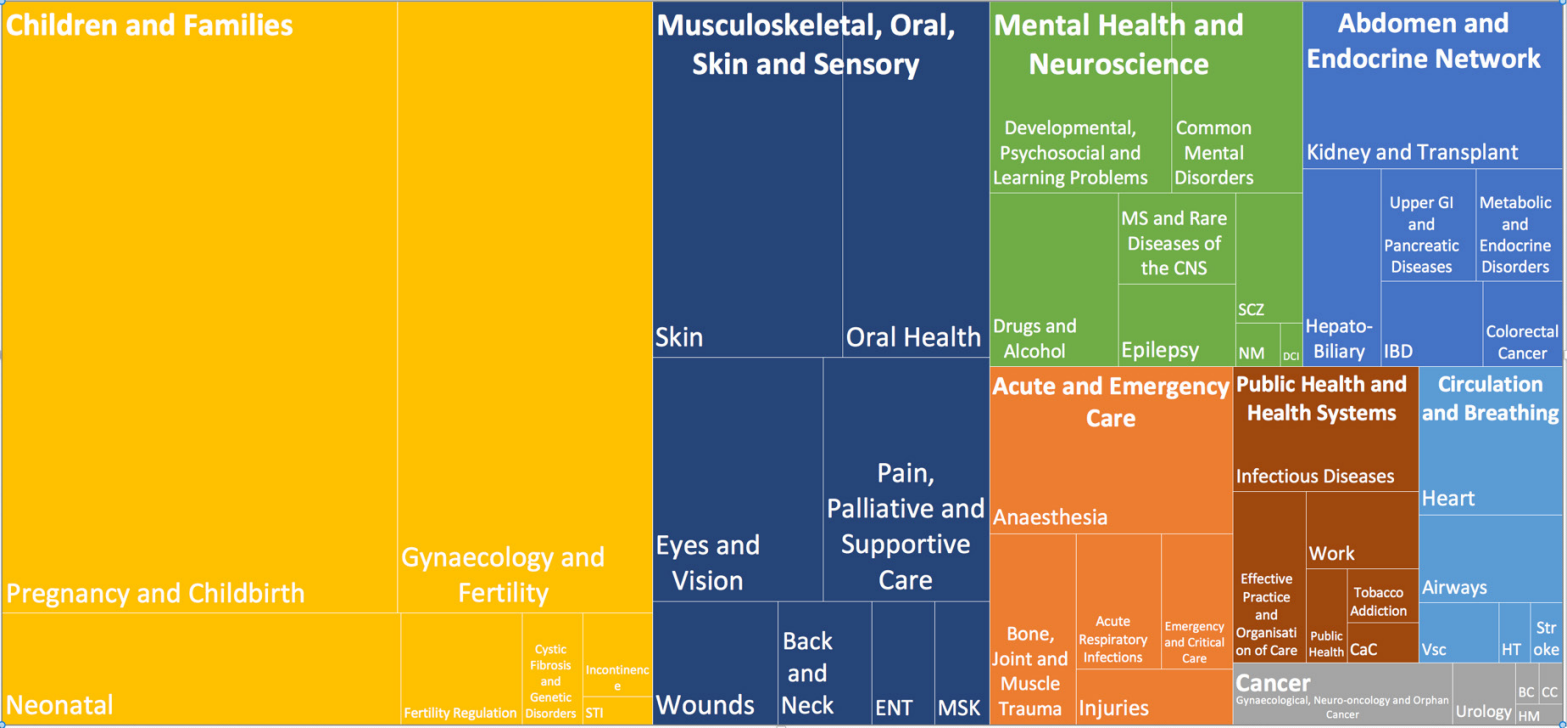
Distribution of Iranian RCTs in the Cochrane reviews based on Cochrane Review Groups. BC, Breast Cancer; CaC, Consumers and Communication; CC, Childhood Cancer; DCI, Dementia and Cognitive Improvement; HM, Haematological Malignancies; HT, Hypertension; MSK, musculoskeletal; NM, Neuromuscular; SCZ, Schizophrenia; Vsc, Vascular.

 The majority (81.0%) of the study Iranian RCTs were performed between 2003 and 2016 in 60 cities. Figure 3 shows the heterogeneous distribution of the location of the centers where the RCTs were performed, with the highest number performed in provinces of Tehran (n = 348, 29.8%), Isfahan (n = 148, 12.7%) and Fars (n = 97, 8.3%). However, reporting was insufficient for the year of conducting the study in 465 studies, the month of beginning of the trial in 283 further studies, the name of the city in 107 studies and the number or name of the centers involved in the trial in 202 studies.

 These studies were published as an article (n = 1149), abstract in conference proceedings (n = 10), IRCT registry (n = 6) and a PhD dissertation. The articles were published in 521 peer-reviewed journals, mostly in English (90.1%) and in domestic journals (33.6%). *Journal of Research in Medical Sciences*, Isfahan University of Medical Sciences (n = 29), the *International Journal of Gynecology & Obstetrics* (n = 29) and the *Iranian Red Crescent Medical Journal* (n = 28) published the highest number of these articles.

 The assignments included an RCT type in 1040 cases (89.2%), a quasi-experimental type in 26 (2.2%) and unclear in one study. Crossover: 21, other: 14, cluster: 7. The treatment (47.3%) and supportive care (24.2%) constituted the most common allocation types.

 The majority of the RCTs’ (83.6%) sample sizes were 30-200 with a median of 80, over 1000 in four of the studies and at most 50 in 25.5%. The Cochrane Airways group reviewed an RCT with a sample size of 12 514 as the maximum and the Public Health group reviewed an RCT with a sample size of 9 as the minimum. The full text of two of the articles was also inaccessible. Table 1 presents the sample size frequency.

**Table 1:** Frequency of Sample Size Groups in Iranian RCTs

**Sample Size Group**	**No. (%)**	**Cumulative**
9–20	34 (2.9)	34 (2.9)
21–40	160 (13.7)	194 (16.6)
41–60	232 (19.9)	426 (36.5)
61–80	176 (15.1)	602 (51.6)
81–100	169 (14.5)	771 (66.1)
101–150	188 (16.1)	959 (82.2)
151–200	87 (7.4)	1046 (89.6)
201–300	59 (5.1)	1105 (94.7)
301–400	29 (2.5)	1134 (97.2)
401–500	9 (0.9)	1149 (98.1)
501–1000	18 (1.5)	1161 (99.6)
1001–12514	5 (0.4)	1166 (100)

 From a methodological perspective, at least one arm was used as the control group in 1077 (92.4%) studies, including 299 (25.6%) that provided this group with a placebo. Moreover, 414 (35.5%) studies were double-blinded, 28 (2.4%) triple-blinded and 266 (22.8%) used no blinding methods in their design.

**Figure 4:**
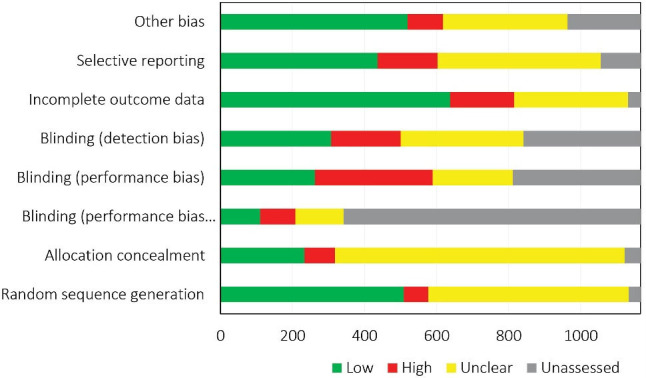
Overall Risk of Bias Assessment of Iranian RCTs Included and Evaluated in Cochrane Reviews.

**Figure 5:**
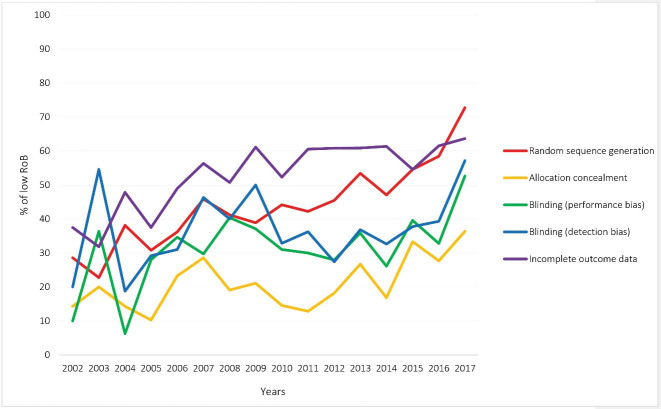
Trend of Low RoB for Four Domains of Cochrane Tool in the Iranian RCTs during 2002–2017.

 Not all the domains of the RoB tool were assessed in all the CRs; for instance, Cochrane reviewers had assessed random sequence generation in 1134 out of the 1166 RCTs and allocation concealment in 1122. Performance and attrition biases respectively received the highest frequency of high RoB (22.9%) and low RoB (56.3%). The RoB of at least one domain was judged as unclear in 931 (79.8%) out of the 1166 included RCTs. Figure 4 shows the RoB assessed in the individual domains of the RoB tool for the included RCTs. According to Figure 5, random sequence generation and incomplete outcome data continuously improved in terms of increasing low RoB during 2002–2017 (The analysis for all domains has been provided in Supplementary file 1).

## Discussion

 This study sought to investigate the quality of Iranian RCTs included in CRs based on Cochrane reviewers’ evaluations. To the best of the authors’ knowledge, this study pioneered RCTs’ quality evaluation at a national scale using the Cochrane RoB tool. The quality of Iranian RCTs was found to be low in terms of the majority of RoB domains with a high or unclear RoB, which were mostly associated with the study design and included random sequence generation, allocation concealment and blinding.

 A few studies mainly assessed RCTs of special subjects or fields using the CONSORT checklist and yielded consistent findings, suggesting the poor methodological quality and reporting of the Iranian RCTs.^21,26-29^

 The present study found the RoB of 44.9% of the Iranian RCTs to be low and 6.0% to be high in terms of random sequence generation. In contrast, the remaining RCTs’ status was impossible to be evaluated in terms of randomization given the inadequate data. Similarly, randomization was invalidated in 35.5%–98.7% of Iranian RCTs owing to their failure to report their randomization method, as was the case for the RCTs conducted in other countries^26,30,31^; for instance, low RoB was reported in terms of randomization for 44% and 62% of RCTs performed in Saudi Arabia^15^ and Sub-Saharan Africa region,^32^ respectively. There are some studies that assessed a large number of RCTs in all subjects and countries. For example, low RoB was also reported for below 50% of 1286 RCTs included in CRs.^33^ Another study with 176,620 RCTs showed that 36.4% and 50.9% of RCTs still suffer from high RoB in random sequence generation and allocation concealment, respectively.^34^ Given that this problem is not specific to Iran, it is recommended that efforts be made at a global scale to enhance the quality of RCTs in this domain.

 Blinding was assessed as two subdomains, i.e., blinding of participants and personnel (performance bias) and blinding of outcome assessors and analysts (detection bias). In terms of performance bias, only 32.3% (n = 262) of the included studies were assessed as low-risk, while 299 studies used placebos as the method of blinding their participants and personnel. This difference between the number of low-risk studies and that of studies using placebos shows Iranian researchers’ failure to comprehend the mechanism of implementing placebos to hide interventions from the participants or indicates their inability to adequately explain the mechanism in a way that Cochrane reviewers are persuaded with the explanation. One-third of the studies were also evaluated as low-risk in terms of detection bias, which can be explained by Iranians’ unwillingness to participate in completely-blinded trials and failure to report the study details, which made it difficult for the reviewers to assess bias. Research generally suggests inadequately-performed blinding in the RCTs conducted in Iran and other countries.^15,33,35^

 Low RoB was assigned to attrition bias in 56.3% of cases, suggesting a small number of patients failing to follow up in the Iranian RCTs. In line with studies assessing Iranian RCTs in this domain, the present study found that Iranian researchers are relatively successful in providing data on the patients withdrawing from the study. Studies conducted in countries other than Iran have also reported the RoB of many RCTs as low in this domain.^15,33^ Low RoB was assigned to selective outcome reporting in 436 (41.3%) RCTs and unclear RoB to 453 (42.9%). This high frequency of unclear RoB can be explained by the failure of the majority of Iranian authors to register their studies in databases such as the IRCT, which resulted in failing to publish the study protocol before the final results were published. The reviewers had therefore, difficulty evaluating this domain based on the outcomes reported in the article. Given the generally difficult assessment of selective outcome reporting and other bias domains,^36^ these results should be cautiously interpreted.

 Unclear RoB is a result of poor reporting. Although poor reporting does not necessarily mean flawed methodology, it hinders adequate assessment of the reliability of the methods and trustworthiness of the results.^37^ A study conducted in 2017 assessed 20 920 RCTs included in CRs in terms of poor reporting and inadequate methods.^38^ When comparing the results of that study with ours, Iranian RCTs had moderately higher unclear RoB in allocation concealment (71.7% vs 57.5%). Besides, unclear RoB was also higher in random sequence generation and incomplete outcome data domains. This reflects the need to train Iranian researchers regarding research methodology and reporting. Furthermore, including a research methodologist should be considered by Iranian research teams.

 In our study, the Iranian RCTs included a small sample, which was below 60 in their majority. The distribution of the sites of conducting the RCTs was also heterogeneous, and many of the studies were unicenter and conducted in Tehran, the capital of Iran, which can be explained by the significantly higher number of universities and top medical universities located in Tehran compared to other cities and provinces. It is recommended that more collaborative, multi-center and high-quality studies be conducted by making appropriate research policies.

 Evaluation of the quality of RCTs in the different field of medical sciences showed the same pattern; for example, only 11.1% of dentistry and oral health RCTs were deemed to be of low overall RoB^39^ or while the quantity of trials in hand surgery has increased over time, the methodological quality has remained low.^40^ Still, with the urgent need for the best available evidence through RCTs during the COVID-19 pandemic, we see the disparity between desired standards and what is done in the real world when conducting and reporting RCTs.^41^

 According to Chalmers and Glasziou, an estimated significant portion (85%) of medical research is wasted in many dimensions and phases, namely relevance of the research question to the patients and physicians, appropriateness of the study design, accessibility of full text and unbiased and usable reporting.^42^ The present findings suggest that the limitations of Iranian RCTs include all these four dimensions. Some of these issues, e.g., low-quality reporting, could be simply avoided. The introduction of the CONSORT statement in 1996^4^ has increased the percentage of studies with low RoB in randomization, blinding of outcome assessors, incomplete data reporting and other sources of bias. However, the high frequency of unclear RoB in all the domains can be an alarming sign of low-quality reporting in Iranian RCTs.

 The strengths of the present study include evaluation of the RCTs by third-party reviewers with no prejudices or bias. This also could be a limitation of this study, as we considered that assessments are correct. RoB was, however, differently reported in different CRs for the same RCT. This discrepancy was also raised in previous assessments of RoB based on the Cochrane tool.^43^

 In conclusions, the present study showed that conducting and reporting of the Iranian RCTs could be improved in several domains. Despite the major improvements observed between the initial and the most recent RCTs, special attention should be paid to methodological training in certain domains such as allocation concealment and blinding. However, this shortcoming has been addressed globally and can be overcome by training and development of interventional policies.

## Supplementary Materials

Supplementary file 1. Time trend for proportion of low, high, and unclear risk of bias in each domain.
