# Supplementary material for: Risk of Bias in Iranian Randomized Trials Included in Cochrane Reviews
Source: Arch Iran Med. 2022 Jun 1;25(6):375–82. doi: 10.34172/aim.2022.61 (PMC11904274; doi:10.34172/aim.2022.61)

Supplementary file 1. Time trend for proportion of low, high, and unclear risk of bias in each domain. (Arch Iran Med. June 2022;25(6))

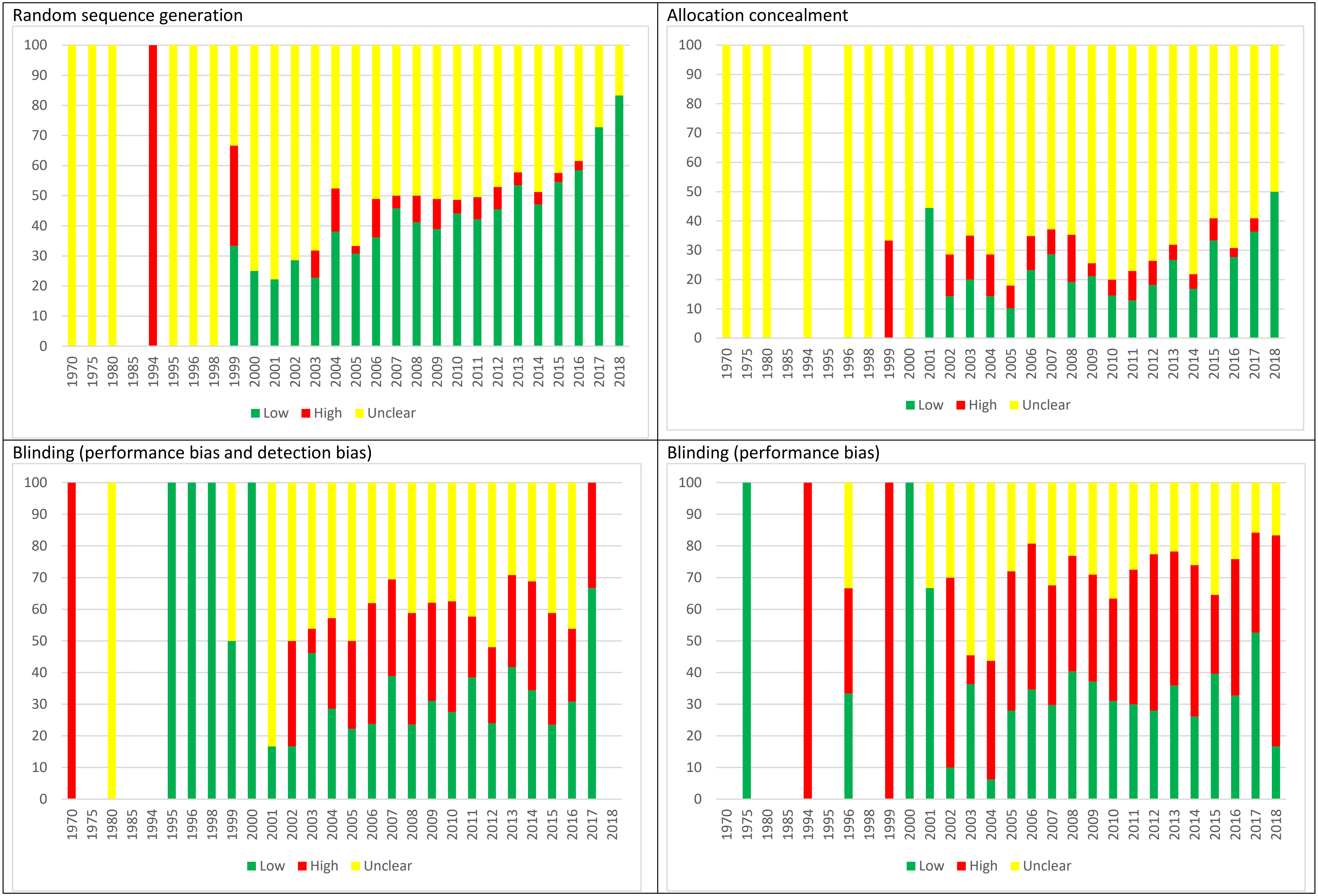

Blinding (detection bias)

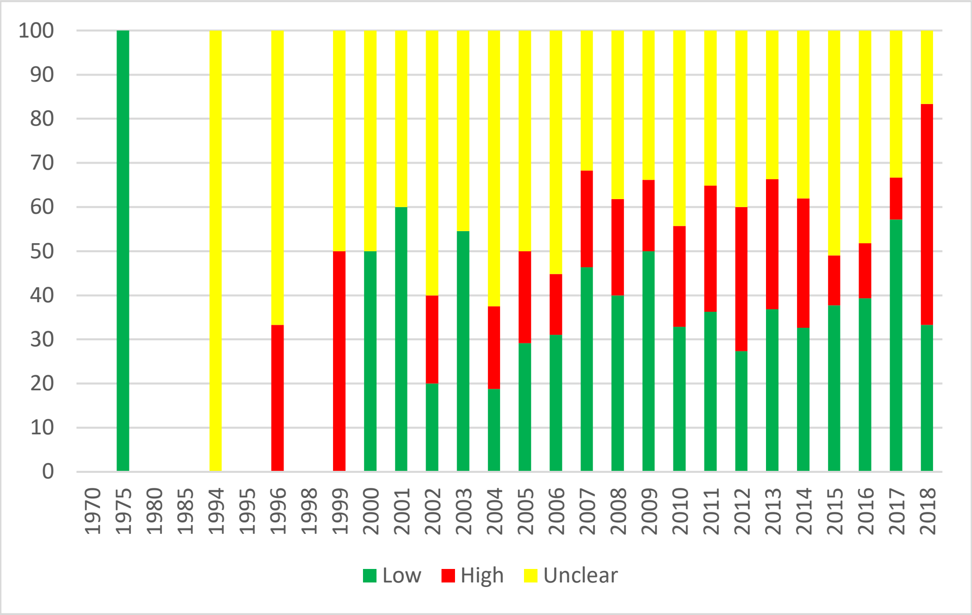

Incomplete outcome data

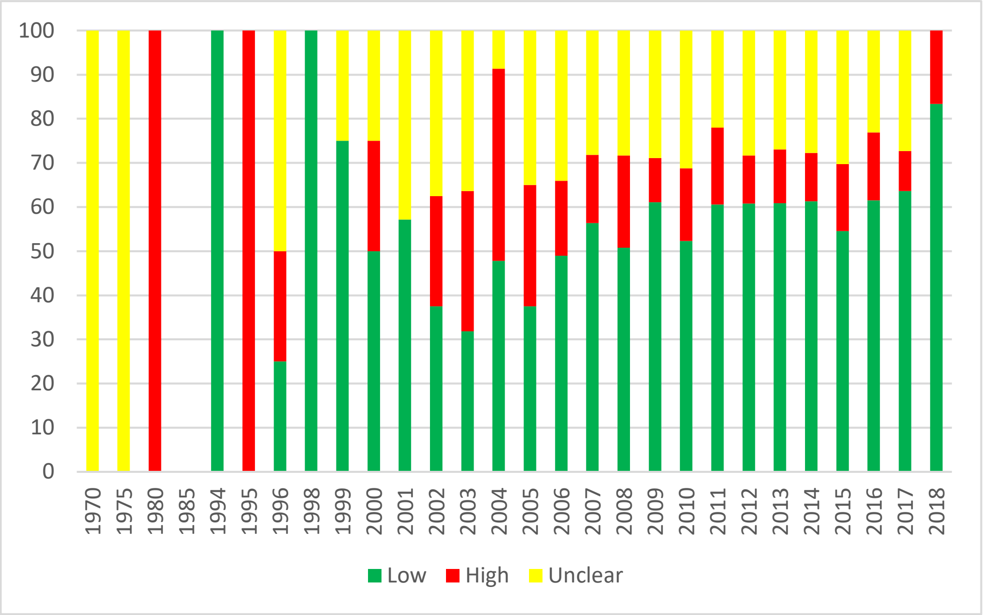

Selective reporting

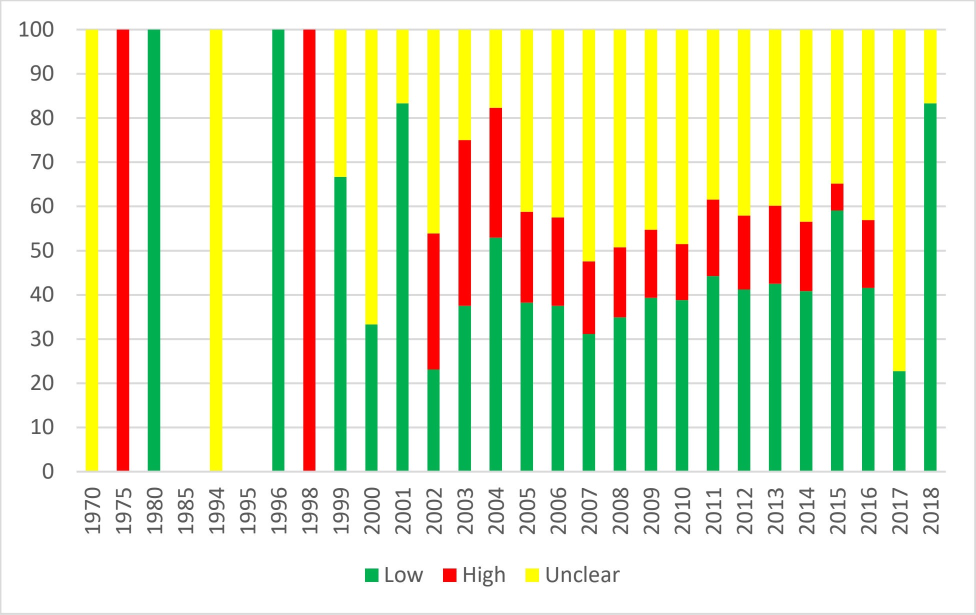

Other bias

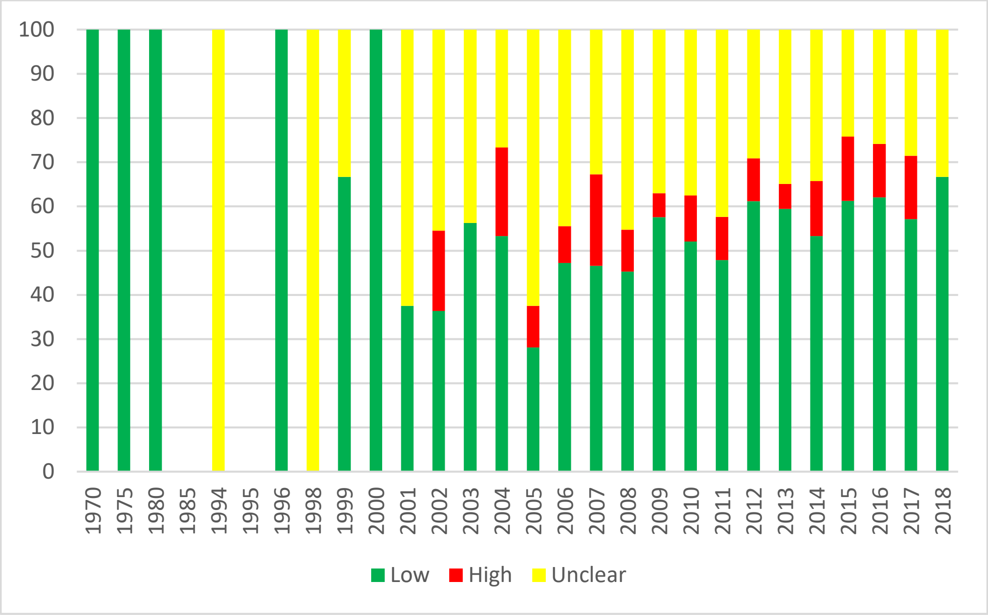

Supplement: Supplementary file 1 — Time trend for proportion of low, high, and unclear risk of bias in each domain. [file aim-25-375-s001.pdf]
